# Exercise‐Based Cardiac Rehabilitation for Patients After Heart Valve Surgery: A Systematic Review and Re‐Evaluation With Evidence Mapping Study

**DOI:** 10.1002/clc.70117

**Published:** 2025-03-25

**Authors:** Rongxiang Zhang, Chenyang Zhu, Shiqi Chen, Feng Tian, Yuan Chen

**Affiliations:** ^1^ Xiamen Cardiovascular Hospital Xiamen University Xiamen China; ^2^ School of Nursing Fujian University of Traditional Chinese Medicine Fuzhou China

**Keywords:** cardiac rehabilitation, evidence mapping, exercise rehabilitation, GRADE, heart valve disease, systematic review

## Abstract

**Objective:**

This systematic review and evidence mapping study aims to assess the effects of exercise‐based cardiac rehabilitation on clinical outcomes in patients after heart valve surgery. By consolidating and visualizing existing evidence, the study seeks to identify gaps in knowledge, evaluate the quality and breadth of current research, and provide guidance for clinical practice and future research. The evidence mapping will highlight under‐researched areas and inform healthcare providers on effective strategies to enhance postoperative recovery.

**Methods:**

A comprehensive search was performed across multiple databases, including PubMed, Embase, Cochrane CENTRAL, Web of Science, CNKI, and Wanfang, up to May 2024. Two reviewers independently screened the articles, extracted relevant data, and assessed study quality. Study characteristics and outcomes were visualized using bubble plots.

**Results:**

Ten systematic reviews/meta‐analyses met the inclusion criteria. Based on AMSTAR‐2, two were rated “high quality,” two “low quality,” and six “very low quality.” Using the GRADE system, of the 48 pieces of evidence across 10 outcomes, 1 was “high quality,” 8 “moderate,” 19 “low,” and 20 “very low.”

**Conclusion:**

Current evidence indicates that exercise‐based cardiac rehabilitation can enhance physical capacity, left ventricular ejection fraction, peak oxygen uptake, and daily living activities in heart valve surgery patients. However, more large‐scale, high‐quality studies are needed to verify its effects on all‐cause mortality, quality of life, adverse events, return to work, and emotional health.

## 1 Introduction

Heart valve disease is a common and serious cardiovascular condition that significantly affects a patient's health, particularly aspects such as cardiac function, exercise capacity, and quality of life. Its incidence increases with age [1, 2, 3]. In 2019, it was estimated that ~40.5 million people worldwide were affected [4]. In recent decades, major advancements in both surgical and interventional techniques have made surgery an important treatment option for many types of heart valve disease [5]. However, many surgical candidates experience reduced cardiorespiratory endurance due to limited physical activity caused by symptoms. While surgery corrects the abnormal valve function, postoperative stress, physical weakness, and prolonged bed rest often lead to further declines in cardiopulmonary fitness [6]. Lack of physical activity, both preoperative and postoperative, is a key factor in increased postoperative morbidity, complications, and longer hospital stays. This significantly impacts patients' recovery, places financial strain on families, and stretches healthcare resources [7, 8, 9].

Exercise‐based cardiac rehabilitation is an essential component of postoperative care following heart valve surgery, with structured exercise rehabilitation programs being a key aspect widely used among cardiovascular patients [10]. Tailored and supervised exercise programs improve exercise capacity, cardiorespiratory fitness, myocardial function, quality of life, and recovery in these patients [11, 12]. Although several systematic reviews have explored exercise‐based cardiac rehabilitation in heart valve surgery patients, existing studies show significant inconsistencies in sample sizes, study designs, and conclusions, which weaken the overall quality and consistency of the available evidence [13]. As a result, the effectiveness and impact of exercise‐based cardiac rehabilitation in this patient group remain insufficiently explored and validated.

Systematic reviews and meta‐analyses play a crucial role in shaping clinical practice and identifying gaps in knowledge, making them essential for evidence‐based healthcare decisions [14]. In comparison, evidence mapping is an innovative technique that systematically gathers, analyzes, and synthesizes existing evidence to highlight areas requiring further research and to set future research priorities [15, 16]. This study aims to assess the impact and effectiveness of exercise‐based cardiac rehabilitation on patients undergoing heart valve surgery through evidence mapping. By systematically reviewing the available literature, this work seeks to build a robust foundation for clinical practice and to identify gaps in current research, guiding future investigations.

## 2 Methods

This systematic review adhered to the Preferred Reporting Items for Systematic Reviews and Meta‐Analyses extension for Scoping Reviews (PRISMA‐ScR) guidelines [17] and followed the methodological framework outlined by the Global Evidence Mapping initiative [18, 19].

### 2.1 Search Strategy

The following English and Chinese databases were searched for relevant studies published up until May 2024: PubMed, Embase, CINAHL, Cochrane Library, CNKI, Wang Fang, CBM, and VIP. The keywords used in the search were: heart valve disease, aortic valve*, mitral valve*, tricuspid valve*, pulmonary valve, valve, exercise therapy, cardiac rehabilitation, endurance training, exercise tolerance, exercise, aerobic exercise, systematic review, and meta‐analysis. The search strategy for PubMed is detailed in Table S1 and was adjusted for each database accordingly. Additionally, the reference lists of included studies were manually reviewed to identify any further relevant reviews.

### 2.2 Inclusion Criteria

#### 2.2.1 Study Type

Systematic reviews or meta‐analyses published in either Chinese or English.

#### 2.2.2 Types of Participants

Participants include those diagnosed with heart valve disease (such as aortic, mitral, or tricuspid valve disease) who have undergone heart valve replacement or repair, either through surgical or interventional methods.

#### 2.2.3 Intervention Type

“Exercise‐based” interventions refer to structured or self‐directed programs conducted in inpatient, outpatient, community, or home settings, encompassing various modes of exercise training [20]. This definition covers a wide range of organized activities aimed at improving physical fitness and overall well‐being, tailored to suit different preferences and environments.

#### 2.2.4 Comparison Type

Standard care or noninterventional approaches that specifically exclude any physical activity components.

### 2.3 Exclusion Criteria

The exclusion criteria were as follows: (i) Full texts that could not be obtained. (ii) Duplicate publications. (iii) Studies with incomplete data: Studies with missing essential data (such as primary outcomes) or insufficient data to draw valid conclusions were excluded. Studies with minor missing data were included if appropriate handling methods, such as imputation, were applied. (iv) Interventions that include exercise programs but where exercise is not the primary intervention.

### 2.4 Literature Screening and Data Extraction

Two independent researchers conducted the literature screening, with discrepancies resolved by a third researcher when necessary. Duplicate records were first removed using literature management software, followed by a review of titles and abstracts to exclude irrelevant studies. The remaining studies were then fully evaluated based on prespecified eligibility criteria. Data extraction was carried out using a customized Microsoft Excel spreadsheet, capturing key information such as publication year, lead author, country of origin, study design, number of studies and participants, interventions, and outcome measures.

### 2.5 Assessment of Study Quality

Two independent evaluators used the AMSTAR‐2 (A Measurement Tool to Assess Systematic Reviews‐2) to assess the methodological quality of the selected systematic reviews and meta‐analyses [21], which includes 7 critical components and 11 additional aspects. Any discrepancies in scoring the 16 items from the AMSTAR‐2 checklist were resolved through discussion between the reviewers, with a neutral arbitrator involved if necessary. The Grading of Recommendations, Assessment, Development, and Evaluation (GRADE) framework [22] was then applied to classify the certainty of the evidence into four levels: high, moderate, low, or very low. Initial ratings could be downgraded due to factors affecting validity, such as bias, heterogeneity, imprecision, inconsistency, or selective reporting [23].

### 2.6 Presentation of Evidence

In this study, we used R software to create a bubble plot, providing a visual overview of the systematic review quality, effect direction, and study types for key outcome measures. The *x*‐axis represents different outcome measures, while the *y*‐axis indicates the quality of systematic reviews based on AMSTAR ratings. The outer color of each bubble denotes the effect direction, the inner color differentiates between randomized controlled trials and nonrandomized studies, and the bubble size reflects the number of studies or sample size. Additionally, we used R software to create a bar chart to display the consistency of conclusions across different systematic reviews for each outcome measure.

In this study, we assessed effect direction following the criteria outlined in the Cochrane Handbook for Systematic Reviews of Interventions. Specifically [24], we classified an effect as positive if the pooled analysis yielded a *p* value < 0.05 and the effect size indicated a favorable outcome (e.g., improvement in outcomes). Negative effects were determined when the pooled analysis yielded a *p* value < 0.05 and the effect size indicated an unfavorable outcome (e.g., increased risk). If the *p* value was ≥ 0.05 or the confidence interval included the null value, the effect was classified as not significant. When no pooled analysis was provided, or there was high heterogeneity or inconsistency among study findings, the effect direction was classified as uncertain. Finally, if no pooled analysis was available but most included studies showed a consistent direction, the effect was categorized as a potential positive or negative effect.

## 3 Results

### 3.1 Study Selection

**Figure 1:**
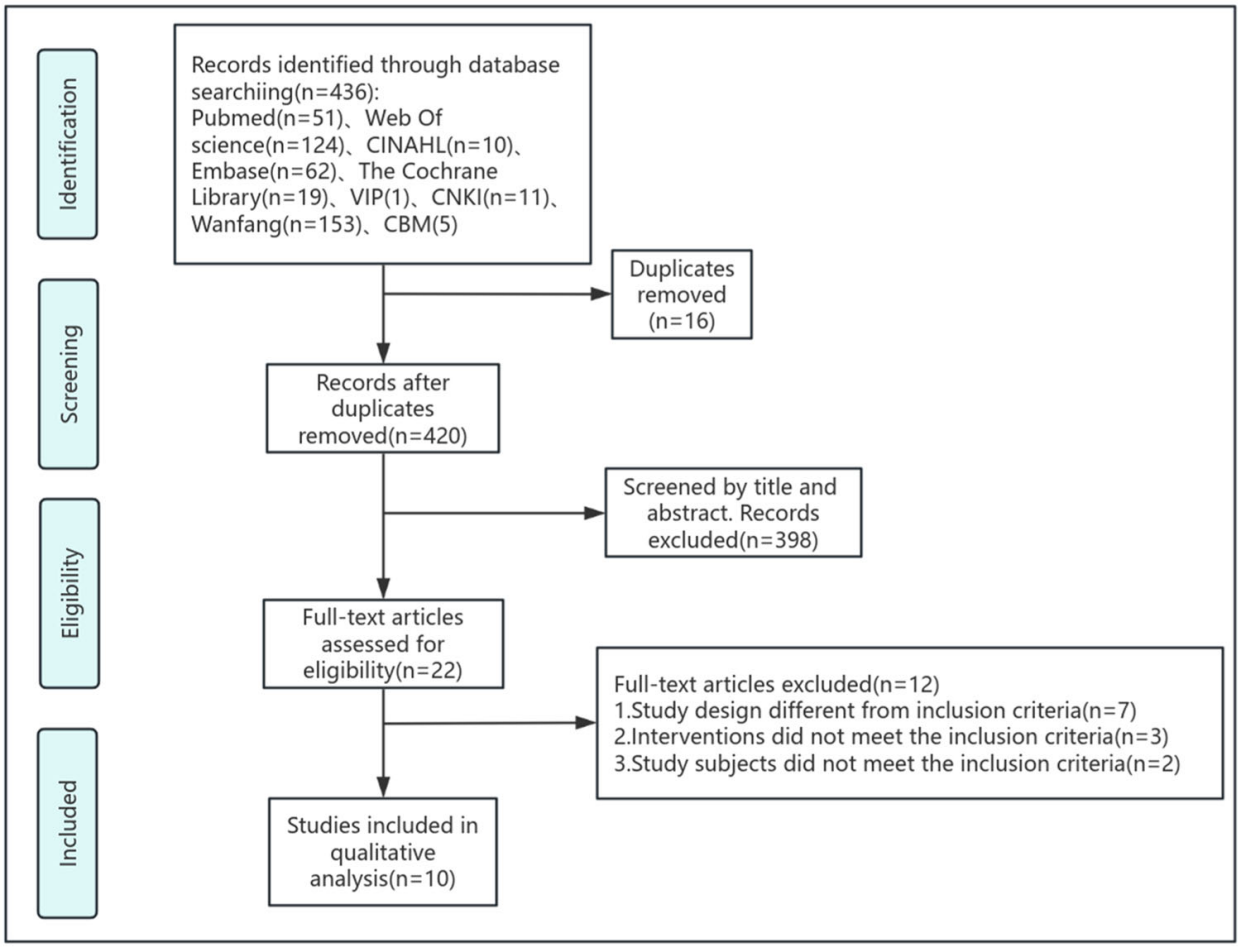
Flowchart of study selection.

Figure 1 provides a visual overview of the literature search process that led to the identification of the eligible studies analyzed in this review. Initially, 436 unique manuscripts were retrieved from electronic databases, with 16 duplicates removed. After screening titles and abstracts, 398 documents were excluded due to irrelevance, leaving 22 potentially suitable studies for full‐text review. Ultimately, 10 articles met the inclusion criteria and were included in the final analysis [20, 25, 26, 27, 28, 29, 30, 31, 32, 33].

### 3.2 Study Characteristics

The summary of the 10 systematic reviews and meta‐analyses included in our study is presented above (Table 1). Of these, two were published in Chinese and eight in English. Four studies (40%) originated from China, while two (20%) were from the UK. Reports from Brazil, Germany, Denmark, and Iran were included in the review. Thirty percent of the reviews included only randomized controlled trials. The number of studies included in each review ranged from 5 to 22, with participant numbers varying between 148 and 2365. Only two systematic reviews or meta‐analyses (20%) reported any exercise‐related adverse effects. Additionally, 50% of the systematic reviews received funding support.

**Table 1:** Inclusion of essential features of systematic evaluation/meta‐analysis (*n* = 10).

References	Country	Trials	Sample size	Types of research designs included in the literature	Treatment intervention	Control intervention	Population	Type of surgery	Does it describe the adverse effects of exercise	Outcomes	Fund
Jinhua et al. [25]	China	11	1569	Randomized controlled studies own before and after controlled studies	Exercise‐based cardiac rehabilitation	Routine care	Patients with transcatheter aortic valve replacement	Interventional	No	a, b	No
Anayo et al. [26]	England	6	314	Randomized vs. nonrandomized controlled trials	Exercise‐based cardiac rehabilitation	Routine care	Surgical aortic replacement and transcatheter aortic valve replacement patients	Interventional and surgical	No	a, e, f, g	No
Sibilitz et al. [27]	Denmark	15	148	Randomized controlled and observational studies	Exercise‐based cardiac rehabilitation	Routine care	Patients undergoing surgery for heart valve disease	Interventional and surgical	No	f, g, h	Yes
Abraham et al. [20]	England	6	364	Randomized controlled and observational studies	Exercise‐based cardiac rehabilitation	Routine care	Patients undergoing surgery for heart valve disease	Interventional and surgical	Yes	a, d, e, f, g, h	Yes
Hosseinpour et al. [28]	Iranian	11	815	Prospective cohort studies, randomized controlled trials, observational studies, retrospective observational, nonrandomized studies	Exercise‐based cardiac rehabilitation	Routine care	Patients with transcatheter aortic valve replacement	Interventional	No	a, b, c, j	No
Oz et al. [29]	German	7	537	Observational and randomized controlled studies	Exercise‐based cardiac rehabilitation	Routine care	Patients with transcatheter aortic valve replacement	Interventional	Yes	a, b	No
Xia [30]	China	12	821	Randomized controlled studies	Sports training	Routine care	Patients undergoing surgery for heart valve disease	Interventional and surgical	No	a, d, e, g, i	Yes
Li et al. [31]	China	12	2365	Observational study	Exercise‐based cardiac rehabilitation	Routine care	Patients with transcatheter aortic valve replacement	Interventional	No	a, b, c, e, j	No
Ribeiro et al. [32]	Brazil	5	862	Observational study	Exercise‐based cardiac rehabilitation	Routine care	Surgical aortic replacement and transcatheter aortic valve replacement patients	Interventional and surgical	No	a, b	Yes
Zeng et al. [33]	China	22	1520	Randomized controlled studies	Exercise‐based cardiac rehabilitation	Routine care	Mitral valve replacement and transcatheter aortic valve replacement	Interventional and surgical	No	a, d, e, i	Yes

*Note:* The study indicates that the number of subjects in this study is the total number of subjects included in the two randomized controlled studies of the study, which did not describe the number of subjects in the 13 observational studies. a, 6‐min walk test; b, activities of daily living score; c, anxiety; d, peak oxygen uptake; e, quality of life; f, return to work; g, adverse event and benefits; h, all‐cause mortality; i, left ventricular ejection fraction; j, depressive.

### 3.3 Results of the Methodological Quality Assessment

We assessed the methodological quality of the 10 systematic reviews using the AMSTAR‐2 tool (Table S2). Notably, only two reviews were rated as “high quality,” while two received “low quality” scores, and half were classified as “very low quality.” Encouragingly, 13 items showed reporting rates above 50%. However, seven reviews did not provide reasons for the selected study types, and another seven failed to disclose funding sources. These findings suggest that there is significant room for improving transparency and methodological rigor in future systematic reviews and meta‐analyses.

## 4 Quality of Evidence Evaluation Results and Effectiveness of Exercise‐Based Cardiac Rehabilitation Interventions

**Figure 2:**
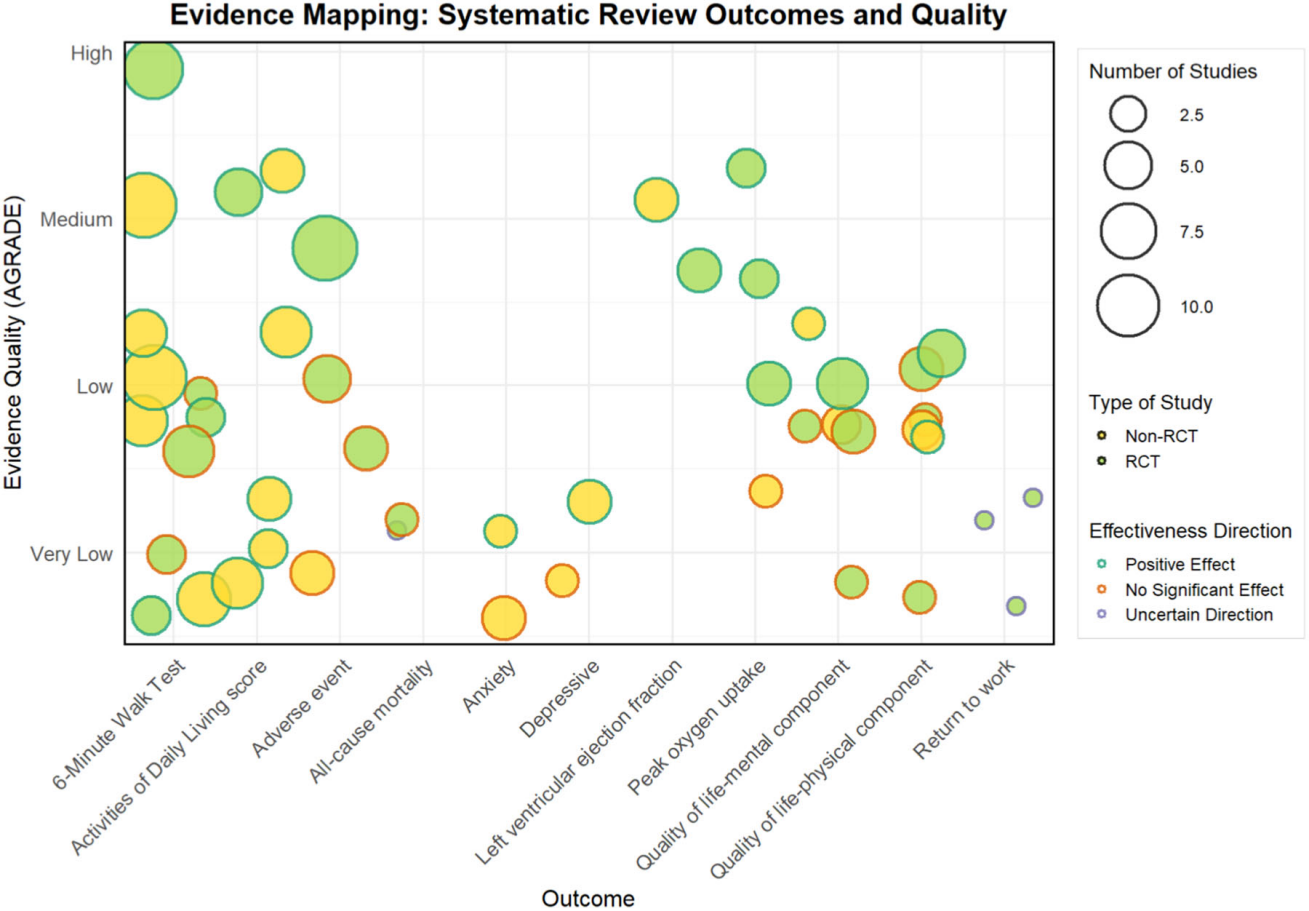
Evidence bubble charts: outcomes and quality of systematic reviews. This bubble chart shows the systematic review results and the quality of evidence measured by outcome. The *x*‐axis lists the results and *y*‐axis shows the quality of evidence (from very low to very high). Bubble size indicates the number of studies, with larger bubbles indicating more studies. The outer color indicates the direction of the effect (dark green: positive effect, yellow: no significant effect, gray: uncertain) and the inner color indicates the type of study (light green: RCT, yellow: non‐RCT).

**Figure 3:**
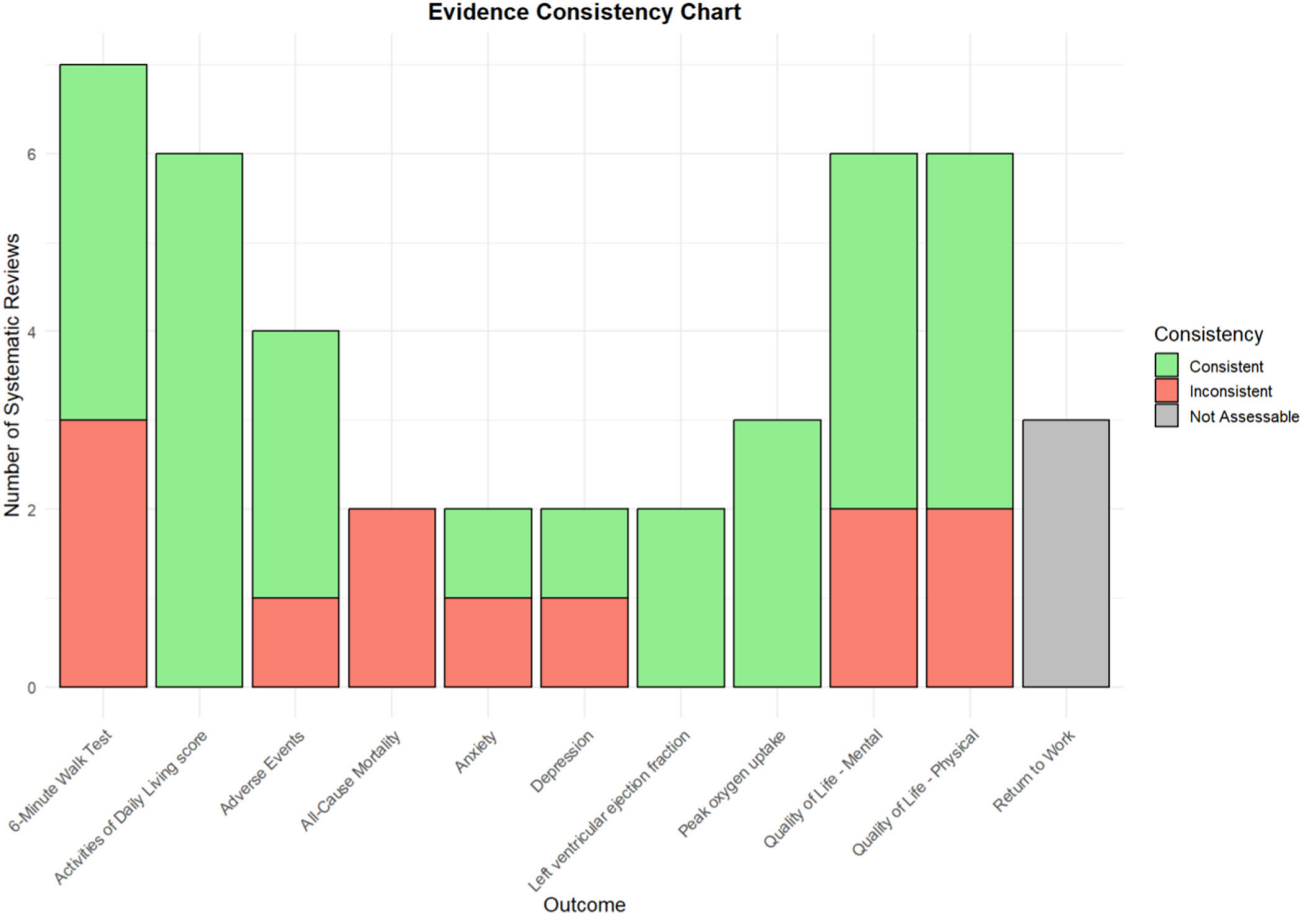
Evidence consistency chart. This bar chart shows the consistency of findings across systematic reviews for each outcome measure. The *x*‐axis lists outcomes, while the *y*‐axis indicates the number of systematic reviews. Green bars represent consistent findings, red bars indicate inconsistent findings, and gray bars denote outcomes that were not assessable due to limited data.

The GRADE evaluation identified 48 pieces of evidence across 10 systematic reviews, covering key outcomes: 6‐min walk test, quality of life, left ventricular ejection fraction, peak oxygen uptake, all‐cause mortality, adverse events, anxiety, depression, and activities of daily living. For more details, refer to Table 2 and Figures 2 and 3.

**Table 2:** Results of evidence evaluation of included studies.

References	Outcomes	Types of combined studies	Trials	Downgrading factors					Upgrading factors	Quality
				Limitations	Inconsistency	Indirectness	Imprecision	Publication bias		
Jinhua et al. [25]	6MWT	R	3	−1	−2	−1	0	0	0	VL
	6MWT	NR	6	−1	0	0	0	0	+1^c^	L
	Activities of daily living score	NR	4	−1	0	−1	0	0	0	VL
Anayo et al. [26]	6MWT	R	2	−2	0	0	0	0	0	L
	Peak oxygen uptake	R	3	−1	0	0	0	0	0	M
	Peak oxygen uptake	NR	2	−1	0	0	0	0	0	VL
	Quality of life—mental component	R	2	−2	0	0	0	0	0	L
	Quality of life—physical component	R	2	−2	0	0	0	0	0	L
	Return to work	R	1	−2	0	0	−1	−1	0	VL
	Adverse event	R	5	−1	0	‐1	0	0	0	L
Sibilitz et al. [27]	All‐cause mortality	R	1	−2	0	0	−1	−1	0	VL
	Adverse event	NR	4	−1	−1	0	−1	0	0	VL
	Return to work	R	1	−2	0	0	−1	−1	0	VL
Abraham et al. [20]	All‐cause mortality	R	2	−1	−1	0	−1	0	0	VL
	Quality of life—mental component	R	2	−1	0	0	−2	0	0	VL
	Quality of life—physical component	R	2	−1	0	0	−2	0	0	VL
	Peak oxygen uptake	R	4	−1	−1	0	0	0	0	L
	6MWT	R	3	−1	−2	0	0	0	0	VL
	Adverse event	R	4	−1	0	0	−1	0	0	L
	Return to work	R	1	−2	0	0	−1	−1	0	VL
Hosseinpour et al. [28]	6MWT	R	3	−1	0	0	−1	0	0	L
	6MWT	NR	11	−1	0	0	0	0	0	L
	Activities of daily living score	NR	4	0	0	0	0	0	+1^c^	M
	Anxiety	NR	4	−1	0	0	0	0	0	VL
	Depressive	NR	4	−1	0	0	0	0	0	VL
	Quality of life—mental component	NR	3	−1	0	0	0	0	0	L
	Quality of life‐physical component	NR	3	−1	0	0	0	0	0	L
Oz et al. [29]	6MWT	NR	7	−1	0	0	0	0	0	VL
	Activities of daily living score	NR	6	−1	0	0	0	0	0	VL
Xia [30]	6MWT	R	6	−1	−1	0	0	0	0	L
	Left ventricular ejection fraction	R	4	−1	−1	0	0	0	+1^c^	M
	Peak oxygen uptake	R	3	−1	0	0	0	0	0	M
	Quality of life‐mental component	R	4	−1	−1	0	0	0	0	L
	Quality of life—physical component	R	4	−1	−1	0	0	0	0	L
	Adverse event	R	11	−1	0	0	0	0	0	M
Li et al. [31]	6MWT	NR	11	0	0	0	0	0	+1^a^	M
	Activities of daily living score	NR	6	0	0	0	0	0	0	L
	Quality of life—mental component	NR	2	0	0	0	0	0	0	L
	Quality of life—physical component	NR	2	0	0	0	0	0	0	L
	Anxiety	NR	2	−1	0	0	0	0	0	VL
	Depressive	NR	2	−1	0	0	0	0	0	VL
Ribeiro et al. [32]	6MWT	NR	5	0	0	0	0	−1	+1^b^	L
	Activities of daily living score	NR	3	0	−1	0	0	−1	0	VL
Zeng et al. [33]	6MWT	R	9	−1	0	0	0	0	+1^c^	H
	Left ventricular ejection fraction	NR	4	−1	−1	0	0	0	+1^c^	M
	Quality of life—mental component	R	6	−1	−1	0	0	0	0	L
	Quality of life—physical component	R	5	−1	−1	0	0	0	0	L
	Activities of daily living score	R	5	−1	0	0	0	0	0	M

*Note:* −1, downgraded by one level; −2, drop two levels; 0, no downgrades and no upgrades; +1, up 1 level.

Abbreviations: 6MWT, 6‐min walk test; H, high; L, low; M, medium; NR, nonrandomized controlled studies; R, randomized controlled studies; VL, very low.

a Large sample size (very large sample size yielded conclusions consistent with other studies).

b There is a clear dose–response relationship (the better the treatment effect, the higher the level of evidence).

c All possible biases tended to weaken the treatment effect in the intervention group but the effect was still clearly effective.

### 4.1 Six‐Minute Walk Test

Seven systematic reviews found that exercise‐based cardiac rehabilitation significantly increased the 6MWT distance for heart valve surgery patients, improving their physical capacity [20, 25, 28, 29, 31, 32, 33]. Of these, one review was rated as high quality, one as moderate, and three as low. However, three reviews reported no statistically significant difference in 6MWT distance between the intervention and control groups [25, 26, 30].

### 4.2 All‐Cause Mortality

Two systematic reviews evaluated all‐cause mortality. One review, rated as very low quality, found no significant difference between the intervention and control groups [20]. The other review included only a single study on mortality and did not perform a meta‐analysis [27].

### 4.3 Adverse Events

Three systematic reviews found no significant difference in adverse event rates between the intervention and control groups, with evidence rated from very low to low quality [20, 26, 27]. However, one additional review, rated as moderate quality, indicated that exercise‐based rehabilitation might reduce the incidence of adverse events [30].

### 4.4 Peak Oxygen Uptake

Three systematic reviews reported significant improvements in peak oxygen uptake in the intervention group compared to controls, with evidence quality rated from low to moderate [20, 26, 30].

### 4.5 Left Ventricular Ejection Fraction

Two systematic reviews showed a significant increase in left ventricular ejection fraction in the intervention group compared to controls, with both reviews rated as moderate quality [30, 33].

### 4.6 Quality of Life

#### 4.6.1 Physical Component

Four reviews found no significant improvement in the physical component of quality of life in the intervention group versus controls, with evidence rated from very low to low [20, 26, 28, 30]. However, two other reviews, rated as low quality, reported improvements in physical quality of life with exercise‐based rehabilitation [31, 33].

#### 4.6.2 Mental Component

Similarly, four reviews reported no significant improvement in the mental component of quality of life, with evidence rated from very low to low [20, 26, 28, 30]. In contrast, two low‐quality reviews suggested improvement in mental quality of life with exercise intervention [31, 33].

### 4.7 Anxiety and Depression

#### 4.7.1 Anxiety

One review found no significant effect on anxiety levels between the intervention and control groups, with very low‐quality evidence [28]. Another review, also rated as very low quality, indicated that exercise‐based rehabilitation might reduce anxiety symptoms [31].

#### 4.7.2 Depression

Similarly, one review found no significant effect on depression [31], while another very low‐quality review suggested improvement in depression symptoms with exercise [28].

### 4.8 Activities of Daily Living and Return to Work

Six systematic reviews consistently reported that exercise‐based rehabilitation significantly improved patients' activities of daily living, with evidence quality ranging from very low to moderate [25, 28, 29, 31, 32, 33]. For return to work, three systematic reviews referenced the same study, which reported that 81% of patients in the exercise group returned to work within 12 months postsurgery, compared to 65% in the control group; however, the study did not report a *p* value [34].

## 5 Discussion

In conclusion, this study underscores the global significance of heart valve disease and highlights the importance of exercise‐based cardiac rehabilitation in improving surgical outcomes for affected patients. Through systematic evaluation and evidence mapping, the study reaffirmed the effectiveness of exercise‐based cardiac rehabilitation in managing heart valve disorders. It also emphasized the need to tailor exercise interventions by adjusting intensity, frequency, and duration to enhance cardiorespiratory function, increase exercise tolerance, accelerate recovery, and reduce economic burdens [12, 35]. While previous research has noted the psychological benefits of cardiac rehabilitation in addressing emotional distress related to cardiovascular surgeries, this study raises questions about similar mental health benefits for heart valve surgery patients [36]. Further research is needed to explore the psychological impact in this specific group. Overall, structured and personalized exercise‐based rehabilitation programs can enhance both physiological and psychosocial outcomes following heart valve surgery [13]. Future research should address unresolved questions about optimal intervention protocols and expand the understanding of patient outcomes beyond traditional cardiovascular measures.

The AMSTAR‐2 tool is a valuable resource for critically assessing the methodological quality of systematic reviews. In evaluating 10 systematic reviews on exercise‐based cardiac rehabilitation for heart valve disease patients, several key challenges were identified: (i) Many studies failed to provide sufficient justification for the inclusion of specific study designs, which compromised the accessibility, reliability, and clarity of their results. Clear descriptions of the study types selected, along with strong rationale, should be part of the inclusion criteria [21]. (ii) Consistent with previous research by Zian et al. [37], numerous studies did not include a comprehensive list of studies excluded during the initial title and abstract screening or provide clear reasons for these exclusions. Maintaining transparent records of all potential inclusions and the reasons for exclusion ensures trustworthiness and accountability. (iii) Many reviews did not disclose the funding sources of the individual studies included, which raises concerns about potential bias and conflicts of interest. Documenting the funding sources in systematic reviews is essential to ensure transparency and credibility.

In summary, current systematic reviews suggest that exercise‐based cardiac rehabilitation may improve outcomes for patients undergoing heart valve surgery. However, the majority of evidence is classified as “low quality” or “very low quality,” mirroring similar findings by Li et al. [38]. Several factors contribute to these suboptimal results: (i) Many original studies had methodological flaws, lacking essential details such as proper random sequence generation, allocation concealment, double‐blinding of investigators and participants, and blinded outcome assessment. Additionally, the nature of exercise rehabilitation makes it difficult to implement blinding for patients and researchers. (ii) High variability among studies, combined with small sample sizes, resulted in wide confidence intervals, which lowered the quality of evidence. The weak evidence from systematic reviews largely stems from significant flaws in primary studies and the lack of large, multicenter, high‐quality randomized controlled trials evaluating exercise‐based cardiac rehabilitation in heart valve surgery patients. To address these gaps, future research should focus on conducting larger, high‐quality original studies, particularly randomized controlled trials, to strengthen the evidence base and better support the role of exercise‐based cardiac rehabilitation in this population.

The evidence‐mapping findings reveal several key gaps in the research on exercise‐based cardiac rehabilitation for patients undergoing heart valve surgery. These gaps include: (i) A lack of focused analysis on surgical procedures for heart valve disease. Existing systematic reviews mainly concentrate on interventional methods like transcatheter aortic valve replacement or hybrid surgical–interventional approaches. (ii) Limited and low‐quality evidence hinders accurate assessment of adverse events, depression, and return‐to‐work outcomes. High‐quality original studies and systematic reviews are needed to explore connections with all‐cause mortality, occupational reintegration, and other relevant aspects. (iii) Current systematic reviews primarily focus on patients with aortic or major heart valve diseases, with insufficient exploration of exercise‐based rehabilitation in mitral and tricuspid valve surgeries. Addressing these gaps would provide a better understanding of responses across different patient groups. (iv) The overall methodological quality of existing systematic reviews remains unsatisfactory. Higher‐quality original studies, along with more rigorous systematic reviews, are essential to strengthen the evidence base for exercise‐based cardiac rehabilitation. Addressing these gaps will improve our understanding, enhance intervention strategies, and support better long‐term outcomes for patients participating in cardiac rehabilitation after heart valve surgery.

Several limitations should be considered when interpreting the results of this study: (i) The inability to combine and analyze effect sizes for outcome indicators may limit a full understanding of the overall impact. (ii) Restricting the literature search to Chinese and English sources introduces potential linguistic bias. (iii) The review includes publications only up to May 2024, which may require future updates to account for newer research. (iv) Significant variations in interventions, durations, and intensities across studies make it difficult to provide detailed descriptions of individual exercise plans.

## 6 Conclusions

Current evidence indicates that exercise‐based cardiac rehabilitation can enhance physical capacity, left ventricular ejection fraction, peak oxygen uptake, and daily living activities in heart valve surgery patients. However, more large‐scale, high‐quality studies are needed to verify its effects on all‐cause mortality, quality of life, adverse events, return to work, and emotional health.

## Ethics Statement

The authors have nothing to report.

## Conflicts of Interest

The authors declare no conflicts of interest.

## Supporting information

Supporting information.

## Data Availability

The original contributions presented in the study are included in the article/supporting material, further inquiries can be directed to the corresponding author.
