# Supplementary material for: Exercise‐Based Cardiac Rehabilitation for Patients After Heart Valve Surgery: A Systematic Review and Re‐Evaluation With Evidence Mapping Study
Source: Clin Cardiol. 2025 Mar 25;48(3):e70117. doi: 10.1002/clc.70117 (PMC11934209; doi:10.1002/clc.70117)
Supplement: Supplementary file 1 — Supporting information. [file CLC-48-e70117-s001.docx]

Supplementary Material

**Supplementary Material Table S1**. Search strategy for the PubMed database.

| Query | Search term |
| --- | --- |
| #1 | “Heart valve disease” [Mesh] |
| #2 | “aortic valve*”[Title/Abstract] OR “mitral valve*”[Title/Abstract] OR “tricuspid valve*”[Title/Abstract] OR “pulmonary valve”[Title/Abstract] OR “Valve”[Title/Abstract] |
| #3 | #1 OR #2 |
| #4 | “Exercise Therapy” [Mesh] OR “Cardiac Rehabilitation”[Mesh] |
| #5 | “[Endurance Training](https://www.ncbi.nlm.nih.gov/mesh/2027863)”[Title/Abstract] OR “Exercise Tolerance”[Title/Abstract] OR “Exercise”[Title/Abstract] OR “Aerobic Exercise”[Title/Abstract] |
| #6 | #4 OR #5 |
| #7 | “meta-analysis”[Publication Type] |
| #8 | “systematic review”[Title/Abstract]OR “meta-analysis”[Title/Abstract] |
| #9 | #7 OR #8 |
| #10 | #3 AND #6 AND #9 |

**Supplementary Material Table S2**. Results of AMSTAR 2 scale evaluations included in the study

| Reviews(Year of publication) | AMSTAR-2 | | | | | | | | | | | | | | | | Quality |
| --- | --- | --- | --- | --- | --- | --- | --- | --- | --- | --- | --- | --- | --- | --- | --- | --- | --- |
|  | 1 | 2* | 3 | 4* | 5 | 6 | 7* | 8 | 9* | 10 | 11* | 12 | 13* | 14 | 15* | 16 |  |
| Guo JH(2021)(25) | Y | N | N | P | Y | Y | P | Y | Y | N | Y | Y | Y | N | N | N | VL |
| Lizette Anayo(2019)(26) | Y | Y | N | Y | Y | Y | P | Y | Y | N | Y | Y | Y | Y | N | Y | L |
| Sibilitz KL(2016)(27) | Y | Y | Y | Y | Y | Y | Y | Y | Y | Y | Y | Y | Y | Y | Y | Y | H |
| Abraham LN(2021)(20) | Y | Y | Y | Y | Y | Y | Y | Y | Y | Y | Y | Y | Y | Y | Y | Y | H |
| Alireza Hosseinpour(2024)(28) | N | Y | N | P | Y | Y | P | Y | Y | Y | Y | Y | Y | Y | N | Y | VL |
| Asena Oz(2023)(29) | N | Y | Y | N | Y | Y | P | Y | Y | N | Y | Y | Y | Y | N | N | VL |
| Xia Q(2021)(30) | Y | N | N | P | Y | Y | P | Y | Y | N | Y | Y | Y | N | Y | N | VL |
| Li, Z(2023)(31) | N | Y | N | Y | Y | Y | P | P | Y | N | Y | Y | Y | Y | Y | Y | VL |
| Ribeiro GS(2017)(32) | N | Y | N | Y | Y | Y | P | Y | Y | N | Y | Y | N | N | N | Y | VL |
| Zeng L(2023)(33) | Y | Y | N | Y | Y | Y | P | Y | Y | N | Y | Y | Y | Y | Y | Y | L |

Y, Yes; P, partial Yes; N, No; VL, Very low; L, Low; H, High; *, denotes a key item
